# Orthosteric muscarinic receptor activation by the insect repellent IR3535 opens new prospects in insecticide-based vector control

**DOI:** 10.1038/s41598-020-63957-x

**Published:** 2020-04-22

**Authors:** Eléonore Moreau, Karolina Mikulska-Ruminska, Mathilde Goulu, Stéphane Perrier, Caroline Deshayes, Maria Stankiewicz, Véronique Apaire-Marchais, Wieslaw Nowak, Bruno Lapied

**Affiliations:** 1 Laboratoire Signalisation Fonctionnelle des Canaux Ioniques et des Récepteurs (SiFCIR), UPRES EA 2647, USC INRA 1330, SFR QUASAV 4207, UFR Sciences, Université d’Angers, 2 boulevard Lavoisier, 49045 Angers, cedex France; 2 Institute of Physics, Faculty of Physics, Astronomy and Informatics, N. Copernicus University, Torun, Poland; 3 Faculty of Biological and Veternary Sciences, N. Copernicus University, Torun, Poland

**Keywords:** Drug discovery, Neuroscience

## Abstract

The insect repellent IR3535 is one of the important alternative in the fight against mosquito-borne disease such as malaria, dengue, chikungunya, yellow fever and Zika. Using a multidisciplinary approach, we propose the development of an innovative insecticide-based vector control strategy using an unexplored property of IR3535. We have demonstrated that in insect neurosecretory cells, very low concentration of IR3535 induces intracellular calcium rise through cellular mechanisms involving orthosteric/allosteric sites of the M1-muscarinic receptor subtype, G protein βγ subunits, background potassium channel inhibition generating depolarization, which induces voltage-gated calcium channel activation. The resulting internal calcium concentration elevation increases nicotinic receptor sensitivity to the neonicotinoid insecticide thiacloprid. The synergistic interaction between IR3535 and thiacloprid contributes to significantly increase the efficacy of the treatment while reducing concentrations. In this context, IR3535, used as a synergistic agent, seems to promise a new approach in the optimization of the integrated vector management for vector control.

## Introduction

Mosquito-biting rates represent a major concern in overall vector capacity. It is possible to drastically lower the spread of mosquito-borne disease by disrupting host-seeking and feeding^1,2^. Therefore, repellents represent an important alternative in the fight against mosquito-borne disease such as malaria, dengue, chikungunya, yellow fever and Zika^3^. Modes of action of the most commonly used insect repellents such as DEET, IR3535, picaridine characterized so far are diverse. They can i) elicit deterrent feeding behavior, ii) modulate mosquito behavior through gustatory mechanism effect *via* gustatory receptor neurons^4,5^ and iii) affect olfactory mechanism of action involving transmembrane odorant receptor proteins^6–8^ located in olfactory receptor neurons^9–17^.

Recent studies indicate that some repellent chemicals, such as DEET can also directly act on both insect peripheral and central nervous systems. They induce locomotor activity disruption, neuroexcitation (*via* octopamine receptors), cholinergic system alterations (e.g., acetylcholinesterase inhibition and M1/M3 muscarinic acetylcholine receptor subtype interactions) and monooxygenase regulation^17–22^. This demonstrates that repellents can modulate multiple physiological functions through complex mechanisms. Unfortunately, the precise mechanisms of how these chemicals modulate the specific molecular targets in insects still remain elusive, contested and/or misunderstood. Exploring precisely the mode of action of such compounds may lead to new more effective alternatives in the Insect Resistance Management for preventing the spread of mosquito-borne diseases. In this context, we commonly use cockroach neurosecretory cells identified as dorsal unpaired median (DUM) neurons to explore the “non-classical” effects of repellents^18,22^. Because DUM neurons are well known in the literature to co-express multiple membrane receptors and/or ion channels targeted by repellents and insecticides, they represent a suitable model to investigate unexpected actions of the repellents^22^. Previous studies performed in DUM neurons, have indicated that mixing low concentration of the repellent DEET, used as a synergistic agent, and the non-pyrethroid insecticide, propoxur, leads to improvement in the effect of propoxur^18^. We have also confirmed that this mixture increases the efficiency of treatments against *Aedes aegypti* mosquitoes *via* a complex calcium-dependent intracellular signaling pathway^18^.

In the present study, we have demonstrated that another repellent, IR3535, induces intracellular calcium rise through novel cellular mechanisms involving orthosteric/allosteric sites of the M1-muscarinic receptor subtype (M1-mAChR), G protein αβγ subunits, background potassium channels and voltage-gated calcium channels. This unexpected mode of action makes IR3535 a suitable candidate as a synergistic agent to increase insect sensitivity to insecticides^23,24^.

## Results

### The insect repellent IR3535 induces multiphasic effects on intracellular calcium concentration

**Figure 1:**
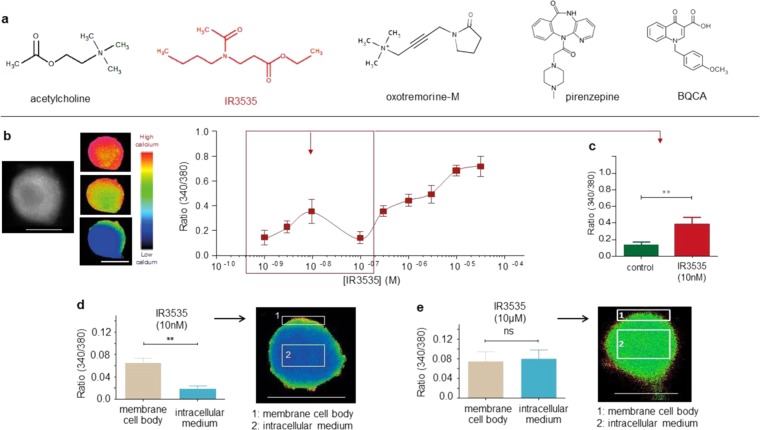
The insect repellent IR3535 induces a non-monotonic concentration-dependent intracellular calcium rise in insect DUM neurosecretory cell body. (**a**) The chemical structure of acetylcholine is shown together with the structure of the repellent IR3535, the selective M1- mAChR subtype agonist oxotremorine-M, the selective M1-mAChR subtype antagonist pirenzepine and the selective positive allosteric modulator of the M1-mAChR subtype (BQCA). (**b**) Bath application of IR3535 produces a multiphasic semi-logarithmic concentration-response curve with the first component reaching a maximum effect obtained at very low concentration (i.e., 10 nM IR3535; square and arrow) as illustrated in comparative histogram in (**c**). (**d**) Images of Fura-2 fluorescence of a single DUM neuron cell body after application of 10 nM IR3535. The elevation in intracellular calcium concentration is measured in parallel at the membrane cell body level (1) and at the intracellular medium level (2). Note that low concentration of IR3535 (10 nM) only induces calcium response in the membrane cell body region. (**e**) Comparative histograms illustrating that higher concentrations of IR3535 (i.e., 10 µM) produce intracellular calcium rise at both membrane cell body and intracellular medium levels. Data are means ± S.E.M. (n = 5); statistical test is Student unpaired t-test **p < 0.01; ns, non significant. Scale bar 55 µm.

The results illustrated in Fig. 1b, suggested an atypical mode of action of IR3535 (Fig. 1a) in isolated DUM neurons. Using calcium imaging, we showed that bath application of IR3535 induced multiphasic intracellular calcium concentration ([Ca^2+^]_i_) rise, depending on the concentrations tested. The mean ratios plotted against the logarithm of the non-cum

ulative concentration of IR3535 revealed an unexpected first component recognized as a « bell-shaped » two processes model involving an increase and a decrease phase between 10^−9^M and 10^−7^M, with a maximum reached at 10^−8^M (Fig. 1b,c). For higher concentrations than 10^−7^M, IR3535 further increased [Ca^2+^]_i_ before reaching maximum effects obtained at 10^−5^M (Fig. 1b). This non-monotonic concentration-response relationship suggested the activation of several membrane and/or intracellular mechanisms involved in the calcium influx and/or calcium release activated by, for instance, upstream G-protein coupled receptors (GPCRs) activation, depending on the concentration of IR3535. After careful observation of the calcium response induced by IR3535, we observed a spatial heterogeneity in the calcium signal according to both the cell region and the concentration of IR3535 (Fig. 1d,e). As illustrated in Fig. 1d, the elevation in [Ca^2+^]_i_ was detected and quantified in two defined fields at membrane cell body level (1) and in the intracellular medium (2). At very low concentration of IR3535 (10 nM), cytofluorescence intensity was only increased at cell membrane level of the DUM neuron whereas there was never significant calcium variation in the intracellular medium (Fig. 1d). By contrast, for higher concentrations of IR3535 (10 µM, Fig. 1e), the intracellular calcium rise occurred simultaneously at cell membrane and intracellular medium regions. This spatiotemporal pattern of calcium signals elicited in DUM neuron indicates that at low concentration of IR3535, the intracellular calcium rise may only imply the presence of a membrane mechanism for calcium influx from the extracellular milieu, whereas at higher concentration, IR3535 may involve additional molecular events such as calcium release from intracellular stores upon GPCRs activation, as previously reported for DEET, in the same preparation^18^. As indicated in the Introduction, for the appropriate use of IR3535 as synergistic agent^24^, we decide, therefore, to only focus on understanding the molecular mechanisms underlying this calcium signal intricacy observed at very low concentrations of IR3535.

**Figure 2:**
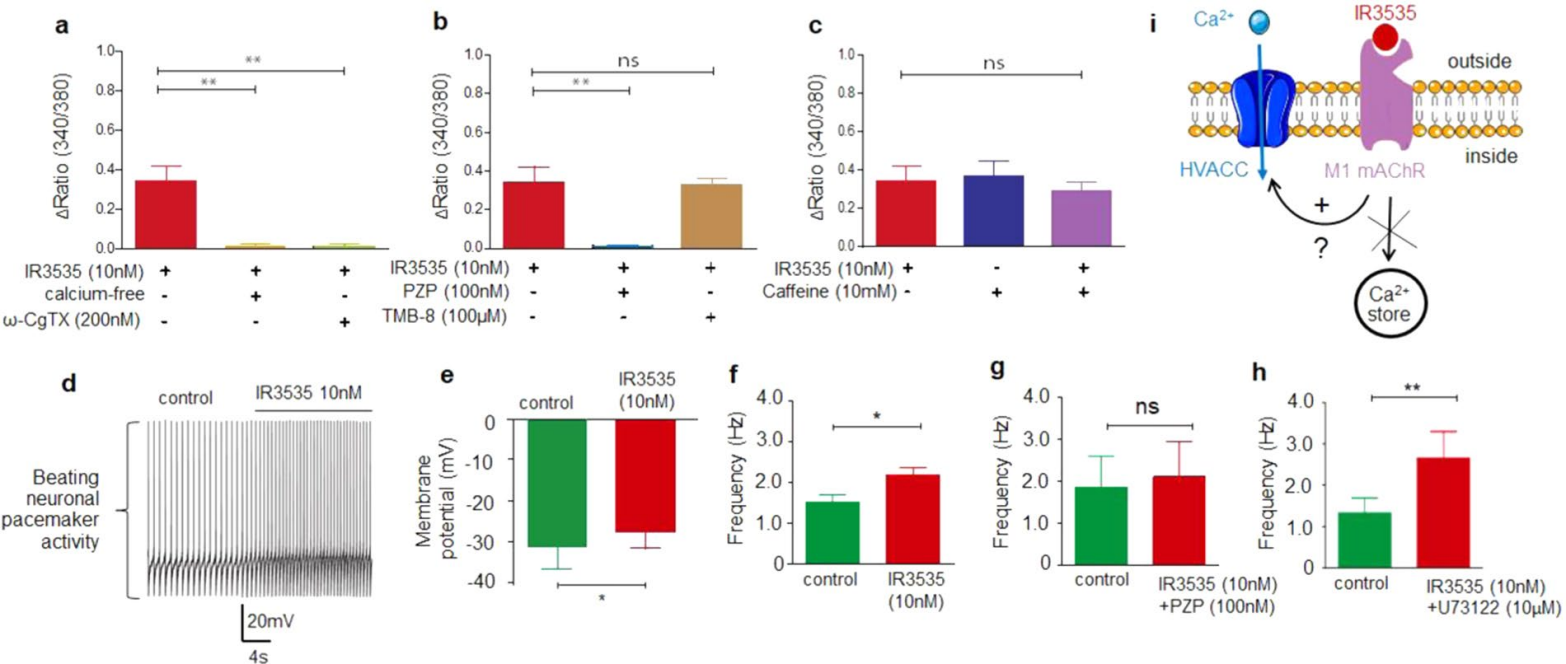
IR3535 increases intracellular calcium concentration through interaction with M1- mAChR subtype and voltage-gated calcium channel activation. (**a**) Histogram summarizing the blocking effects of the pretreatment with EGTA-buffered calcium-free solution and ω-CgTX GVIA, the potent N-type high voltage-activated calcium channel inhibitor, on IR3535-induced elevation of intracellular calcium concentration. (**b,c**) illustrate the inhibitory action of pirenzepine (PZP, (**b**) the selective M1-mAChR subtype antagonist, on intracellular calcium rise produced by IR3535 whereas TMB-8, the IP3 receptor inhibitor (**b**) and caffeine (**c**) do not produce any effect on the intracellular calcium elevation. (**d**) is a representative example of the DUM neuron cell body beating pacemaker activity recorded using the whole-cell patch-clamp technique in control and in the presence of bath applied low concentration of IR3535 (10 nM). (**e,f,g**,**h**) Comparative histograms showing the effects of IR3535 (10 nM) on different beating pacemaker electrophysiological parameters including membrane potential (**e**), action potential discharge frequency in control (**f**), with pirenzepine (PZP) (**g**) and with U73122, an inhibitor of PLC (**h**). (**i**) Hypothetic model illustrating the participation of the membrane molecular events identified as M1 muscarinic acetylcholine receptor (mAChR) subtype and high voltage-activated calcium channels (HVACC) involved in the intracellular calcium elevation induced by the insect repellent IR3535 used at low concentration (10 nM). Average data is shown as mean ± S.E.M. (n = 6); statistical test is Student unpaired t-test **p < 0.01; *p < 0.05; ns, non significant.

We next examined the source of intracellular calcium rise observed in DUM neurons. When the experiments were performed in an EGTA-buffered calcium-free superfusing solution, the IR3535-induced elevation in [Ca^2+^]_i_ was completely abolished (Fig. 2a). It is known that calcium influx in DUM neurons can be accomplished by high voltage-activated-calcium channels (HVACC)^25,26^. Then, additional experiments were performed with ω-conotoxin GVIA, a potent N-type HVA calcium channel blockers. As shown in Fig. 2a, 200 nM ω-conotoxin GVIA produced similar effects to those obtained with the EGTA-buffered calcium-free solution. These results indicate that the intracellular calcium rise results from influx of extracellular calcium through plasma membrane calcium channels. Previous findings reported that low concentration of the repellent DEET induced changes in [Ca^2+^]_i_ *via* activation of M1-mAChRs^18^. Pirenzepine, the selective M1-mAChR antagonist was therefore tested. As illustrated in Fig. 2b, 100 nM pirenzepine totally blocked the IR3535-induced calcium rise. Finally, M1-mAChRs are known to be mainly coupled to phospholipase C (PLC) second messenger pathway. To examine if the effect of IR3535 was related to the downstream activation of inositol triphosphate (IP3) receptors, which thereby increases intracellular calcium concentration, TMB-8 (100 μM), an IP3 receptor antagonist, was tested. TMB-8 did not affect the intracellular calcium elevation produced by 10 nM IR3535 (Fig. 2b). In addition, caffeine (10 mM), known to stimulate the release of calcium from internal stores, did not produce any effect on the IR3535-iduced calcium rise (Fig. 2c). These results suggest the existence of an unusual signaling mechanism between M1-mAChR activated by a low concentration of IR3535 that can stimulate intracellular calcium rise.

We decided to further elucidate the signal transduction leading to IR3535-iduced calcium rise using the whole-cell patch-clamp technique. All DUM neuron tested in this study generated endogenous beating pacemaker activity. The overshooting spikes were separated by a slow predepolarization phase, during which the threshold for action potential triggering was reached, with a spontaneous frequency ranging from 1.5 to 2 Hz, at room temperature (Fig. 2d). Bath application of 10 nM IR3535 produced a slight depolarization (3–5 mV; Fig. 2e) associated with a significant increase in the action potential discharge frequency (Fig. 2f). The blocking action of 100 nM pirenzepine (Fig. 2g) and the lack of effect of U73122 (10 µM; Fig. 2h), which is an inhibitor of PLC-dependent processes triggered by M1-mAChR activation together with previous results (Fig. 2a,b,c) confirm the involvement of M1-mAChRs and the absence of classical signaling pathway activation (Fig. 2i).

### Gβγ subunits relay signal from M1-mAChR orthosteric site to background potassium channels

**Figure 3:**
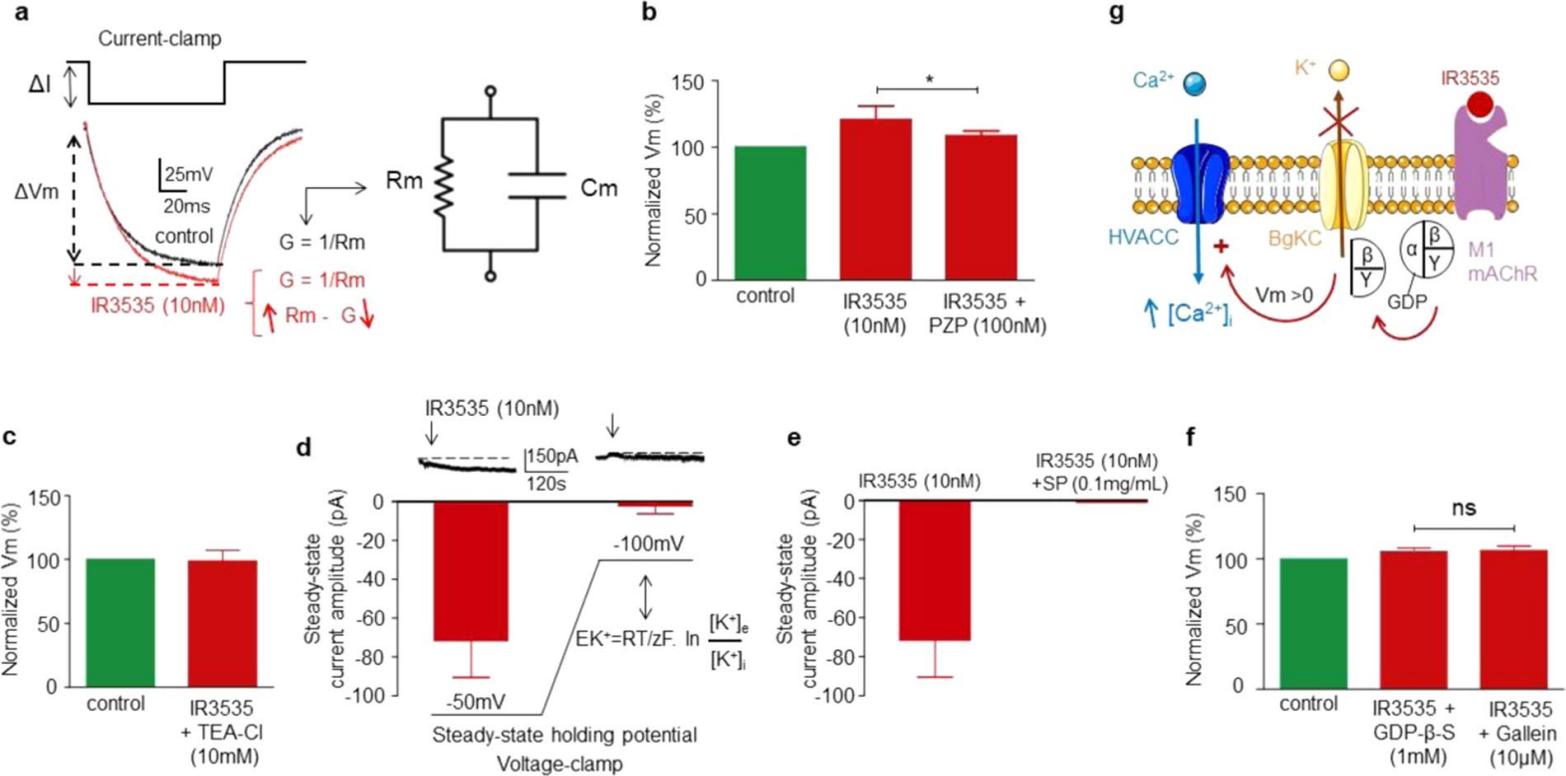
Involvement of the background potassium channel inhibition through a Gβγ-dependent mechanism in the IR3535-induced calcium rise. (**a**) IR3535 (10 nM) induces an increase of the input membrane resistance studied under current-clamp in response to a 100-ms hyperpolarizing current pulse. Reduced equivalent circuit of the DUM neuron cell body plasma membrane obtained by combining a fixed capacitance (Cm) in parallel with ion-specific pathway (Rm). The reciprocal of electrical membrane resistance (Rm) is conductance (G). If the resistance to movement of ion across the membrane is high then the conductance of the membrane to this particular ion is low. (**b**) Histogram summarizing the membrane potential recorded in response to a hyperpolarizing current pulse (100 ms in duration) in control and in the presence of IR3535 (10 nM) and pirenzepine (PZP). (**c,d**) Involvement of background potassium channels (BgKC) in the effect of IR3535. IR3535-induced increase of the input membrane resistance is completely inhibited by the potassium channel blocker TEA-Cl (**c**). Because BgKC, open at the resting state (i.e., −50mV), mediates a permanent spontaneous outward potassium conductance, the resulting steady-state inwardly directed current recorded, under voltage-clamp, in the presence of IR3535 (10 nM) correlate well with the switch from the open to closed state of potassium channels (**d**). (**d,e**) This inward current amplitude is strongly reduced when the holding membrane potential is hyperpolarized to −100mV, which corresponds to the calculated Nernstian equilibrium potential for potassium ions (−100.8 mV, in our experimental conditions) (**d**) and in the presence of SP-related peptide (0.1 mg/mL), known to inhibit binding of G proteins to mAChR (**e**). (**f**) Histograms illustrating the involvement of Gβγ protein in IR3535-induced increase of the input membrane resistance recorded following intracellularly applied GDP-β-S and gallein, a specific Gβγ dimer signaling inhibitor. (**g**) Hypothetic scheme illustrating the participation of the molecular events involved in the effect of IR3535 *via* M1 muscarinic acetylcholine receptor subtype (M1-mAChR) activation. IR3535 induces inhibition of BgKC through Gβγ-dependent mechanism. This produces membrane depolarization (Vm > 0) and activation of high voltage-activated calcium channel (HVACC), resulting in the intracellular calcium rise. Individual controls were normalized as percentage of control. Data are means ± S.E.M. (n = 5); statistical test is Student unpaired t-test *p < 0.05; ns, non significant.

DUM neurons are characterized by a membrane potential depending on the external concentration of both potassium and sodium^25,27^. We previously illustrated that IR3535 (10 nM) induced depolarization associated with an increase in the action potential discharge frequency (Fig. 2d,e,f). To ensure that the depolarization observed was due to an activation or an inhibition of the resting conductances, we tested the effect of 10 nM IR3535 on the DUM neuron input membrane resistance, under current-clamp condition (Fig. 3a). IR3535 produced a significant increase in the input membrane resistance in response to a hyperpolarizing current pulse (100 ms in duration; Fig. 3a,b), which was completely blocked by pirenzepine (100 nM; Fig. 3b). This indicated that the depolarization mediated by M1-mAChR activation was due to the loss of a hyperpolarizing resting conductance. In other words, the inhibition of background potassium channels (BgKC) involved in the maintenance of the membrane potential could account for the membrane depolarization observed in the presence of IR3535. This was substantiated by two sets of experiments performed in both current- and voltage-clamp. First, the IR3535-induced increase in the input membrane resistance was inhibited in the presence of the potassium channel blocker TEA-Cl (10 mM; Fig. 3c). Second, voltage-clamp experiments performed at a steady-state holding potential of −50mV revealed that application of IR3535 (10 nM) generated an inward current, which completely disappeared when a steady-state hyperpolarization imposed upon the DUM neuron to bring its resting potential to −100 mV. A value, which was very close to the calculated Nernstian equilibrium potential for potassium ions (−100.8 mV, in our experimental condition; Fig. 3d). In fact, and according to the results presented in Fig. 3a,c, the inwardly directed current observed after IR3535 (10 nM) correlated well with the inhibition of the spontaneous potassium conductance and the consecutive increase in input membrane resistance. These results confirm that IR3535, acting through activation of M1-mAChR, is able to inhibit BgKC, which thereby induce depolarization capable of activating calcium influx *via* HVA calcium channels (Fig. 3g).

The question that still arised was to determine how M1-mAChRs activated by low concentration of IR3535 were connected to BgKC. It is well recognized that ligand binding to M1-mACh receptors initiates G protein-dependent activation of PLC and consequent generation of IP3 and diacylglycerol. We demonstrated that this signaling pathway was not activated in the presence of 10 nM IR3535 (Fig. 2). However, G proteins can also act on ionic channels in a direct manner^28^. We next focused on the characterization of the cellular mechanisms involved in the direct G protein-dependent modulation of BgKC activity following activation of M1-mAChR. Experiments were performed using [D-Trp7,9,10]-substance P, a substance P-related peptide (SP) that inhibits binding of G proteins to mAChR. As shown in Fig. 3e, SP (0.1 mg/mL), intracellularly applied through the patch pipette, completely blocked the IR3535-induced inward current recorded at a steady-state holding potential of −50 mV. In addition, experiments were performed under current-clamp, using GDP-β-S, a non-hydrolysable GDP analog (Fig. 3f). It is assumed that in the absence of receptor stimulation, the heterotrimeric G protein Gα and Gβγ subunits remain associated in a GDP-bound, an inactive form physically dissociated from the receptor. IR3535 binding to the M1-mAChR should initiate conformational changes allowing coupling with Gαβγ. This would promote a conformational change in Gα, which induces GDP release. In this case, GTP binds to Gα and GDP/GTP exchange drives the dissociation of Gα from Gβγ, switching its conformation to the active state. As shown in Fig. 3f, GDP-β-S (1 mM), intracellularly applied through the patch pipette, completely blocked the increase in the imput membrane resistance produced by IR3535 (Fig. 3b). All together, these results indicate that inhibition of BgKC by M1-mAChR activation could involve G protein and subsequent Gα and Gβγ subunits dissociation. We previously reported that Gα activation was not involved in the IR3535-induced intracellular calcium rise (Fig. 2b,c,h). Therefore, we examined whether increase in the imput membrane resistance can be blocked by pretreatment with a Gβγ blocker, gallein^29–31^. Interestingly, similar results to those of GDP-β-S were obtained with gallein (10 µM; Fig. 3f). Based on these results, we propose that the release of Gβγ following M1-mAChR orthosteric site activation by IR3535 (10 nM) mediates the inhibition of the BgKC, which thereby initiate depolarization necessary to produce calcium influx through HVA calcium channel activation (Fig. 3g).

**Figure 4:**
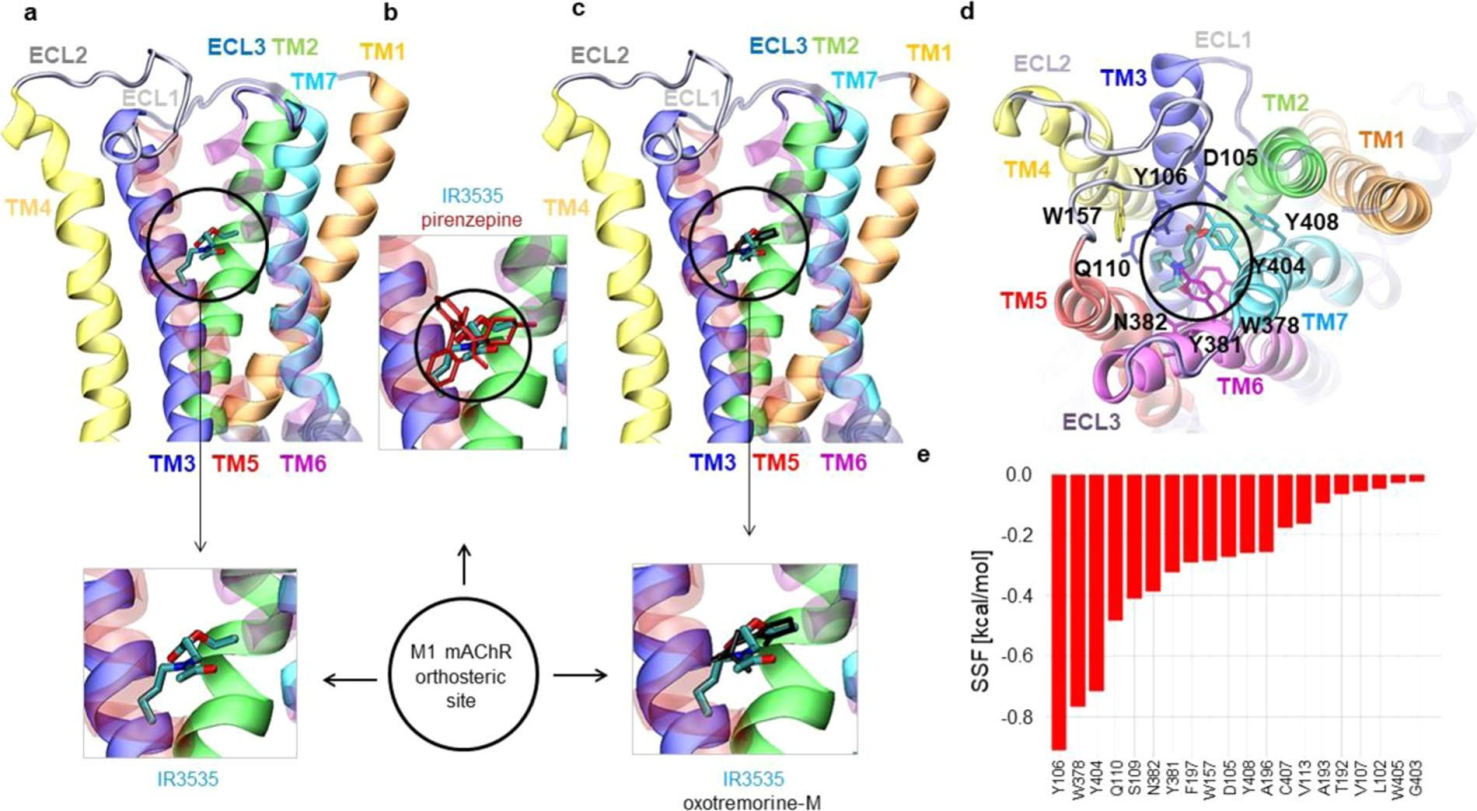
Computational docking results of ligands interactions at the orthosteric site of M1 mACh receptor. The highest affinity poses of: (**a**) IR3535 (black circle), (**b**) pirenzepine (red sticks) and (**c**) oxotremorine-M (black sticks) located at the orthosteric site of M1-mAChR. The mean value and standard deviation of binding energy are calculated for each ligand based on the five runs. The energy of binding is respectively: (−6.2 ± 0.4) kcal/mol for IR3535, (−7.9 ± 0.4) kcal/mol for pirenzepine and (−5.9 ± 0.6) kcal/mol for oxotremorine-M. (**d**) Close-up view of the highest affinity pose of IR3535 from the *top view* of M1-mAChR. Residues observed in the close vicinity of IR3535 are displayed in stick representation and coded by the color of transmembrane helices: TM1 - orange, TM2 - green, TM3 - dark blue, TM4 - yellow, TM5 - red, TM6 - magenta, TM7 - light blue. (**e**) Residues with the highest contribution to the energy of binding of IR3535 at the orthosteric site of M1-mAChR.

Based on experiments illustrated in Figs. 2 and 3, it is suggested that IR3535 used at low concentration (10 nM) interacts with M1-mAChR orthosteric site. To substantiate this hypothesis, computational molecular docking of IR3535 together with pirenzepine and oxotremorine-M, known to act on M1-mAChR orthosteric site, was performed (Fig. 4; Supplementary Figure 1a). M1- mAChR is a bundle of seven-transmembrane α-helices. Their transmembrane central parts form a broad cavity capable of adopting acetylcholine or other relatively small ligands. The volume of this pocket, estimated by using POCASA server^32^ is 3640 Å^3^ (PDB code: 5cxv). Ligands can affect the receptor structure and dynamics by binding to the orthosteric (OS) and/or allosteric site (AS). In order to gain structural insights into the receptor-ligand interactions and explain the *in vitro* observed effects, we examined the interactions of several ligands: (i) IR3535 (ii) pirenzepine, (iii) oxotremorine-M (see Fig. 1a and Supplementary Figure 1b, c, d), with M1-mAChR. The strength of interactions of IR3535 with the M1-mAChR at the OS (Fig. 4a,d), were estimated as a calculated value of SFF parameter. These computed interactions, were found to be stronger in OS than in the AS (see Fig. 7a,c), −6.2 *versus* −5.1 kcal/mol. Of particular interest were three residues in OS, Y106, W378 and Y404, located in the close proximity of IR3535 (Fig. 4d,e; Supplementary Figure 1a), which gave the highest contribution to the energy of binding (Fig. 4e). Also a high score was calculated for Q110, S109, N382, Y381, F197, D105, W157, Y408, and A196 which contributed to the compound’s stabilization. Other residues located within a 6 Å distance from IR3535 did not contribute to the SSF substantially (Fig. 4e). The orthosteric site pose of IR3535 was in a good agreement with the highest affinity pose of pirenzepine (Fig. 4b, red sticks) and oxotremorine-M (Fig. 4c, black sticks). The calculated binding affinity of oxotremorine-M to M1-mAChR was comparable to IR3535 whereas the binding energy of pirenzepine was almost twice as high. Notably, IR3535 binding results in 8% reduction of the OS estimated volume, while pirenzepine occupied more space and this reduction was 16% (Supplementary Figure 1b, c, d). It should be noted that these detailed computer modeling results can not be compared with radiolabeled ligands binding due to the lack of experimental data.

### Bell-shaped concentration-effect relationships of IR3535, recorded at low concentrations, result from an interaction with a M1-mAChR allosteric site

**Figure 5:**
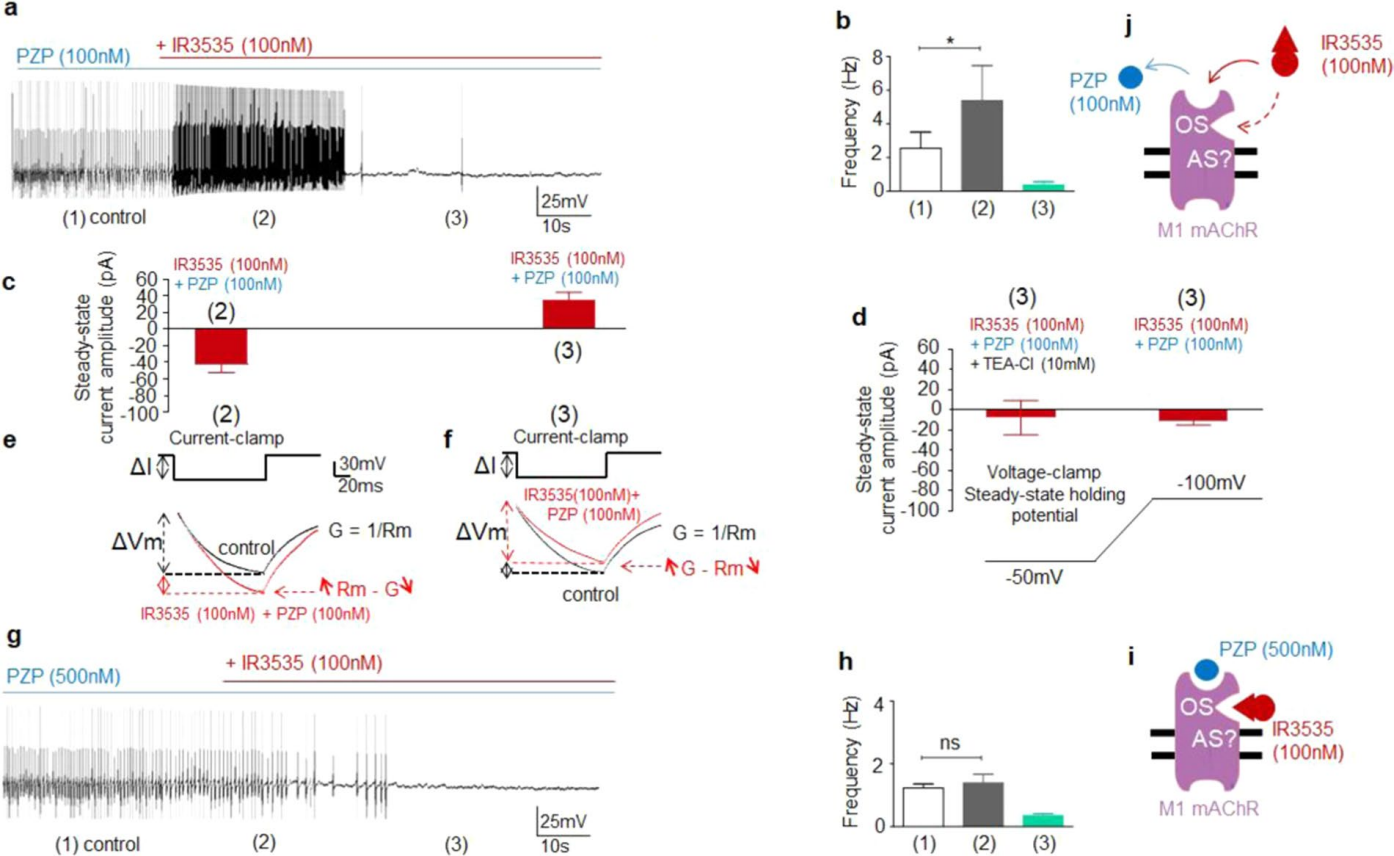
High concentration of IR3535 generates outward potassium current following the early inward current. (**a**) Under current-clamp, in control condition (1) and in the presence of pirenzepine (PZP,100 nM), IR3535 (100 nM) produces a dual effect on DUM neuron action potential discharge frequency. The first effect corresponds to an increase of the action potential frequency (2), followed by an important reduction (3). (**b**) Histogram representing the dual effect of IR3535 (100 nM) on spontaneous electrical activity, recorded at different times of exposure, as indicated. (**c**) Under voltage-clamp, at a holding potential of −50mV and in the presence of pirenzepine (PZP, 100 nM), IR3535 (100 nM) induces an early steady-state inward current (2) followed by an outwardly directed current (3). (**d**) The outward current (3) is reduced in the presence of TEA-Cl (10 mM) and when the resting membrane potential is hyperpolarized to −100mV, corresponding to the calculated Nernstian equilibrium potential for potassium ions (−100.8 mV). (**e,f**) IR3535 (100 nM) induces an increase (2) and a decrease (3) in the input membrane resistance studied under current-clamp in response to a 100-ms hyperpolarizing current pulse. Both inward (2) and outward (3) currents correlate well with the increase (2) and decrease (3) in the input membrane resistance, which thereby influence the action potential discharge frequency. (**g**) Spontaneous action potentials recorded in the presence of pirenzepine (PZP; 500 nM) and after bath application of IR3535 (100 nM). (**h**) Histogram illustrating the effects of bath application of IR3535 (100 nM) on DUM neuron cell body action potential discharge frequency, pretreated with PZP (500 nM; (1) control). After 15 s of exposure, the pacemaker activity decreases (2) and then finally stoppes (3). The increase in the action potential discharge frequency is never observed with PZP used at 500 nM. Data are shown as mean ± S.E.M. (n = 6–8), statistical test is Student unpaired t-test *p < 0.05; ns, non significant. (**i,j**) Schemes suggesting that high concentration of IR3535 could interact with two distinct sites (i.e., orthosteric (OS) and putative allosteric (AS) sites) on M1-mAChR. In addition, high concentration of IR3535 (100 nM) can displace PZP from orthosteric site (**j**) indicating that more concentration of PZP is required to get full effect on orthosteric site (**i,j**).

The bell-shaped concentration-effect relationships (Fig. 1b) might be analyzed under the assumption that M1-mAChR could have a high affinity activation site and a low affinity « inhibition » site. A possible explanation for this bell-shaped curve would be the presence of an « inhibitory » component activated by higher concentration of IR3535 (i.e., 100 nM). This hypothetical « inhibitory » component could be mediated *via* the same M1-mAChR that modulates the stimulatory component, meaning the involvement of a M1-mAChR allosteric site. We therefore examined the effects of 100 nM IR3535 under current-clamp. Experiments performed with pirenzepine used at 100 nM revealed a dual effect of IR3535 (100 nM; Fig. 5a) dissected into two main phases. The first phase corresponded to an increase in the action potential discharge frequency induced by IR3535 (100 nM) (Fig. 5a(2),b(2)), resulting from the slight depolarization (3-4 mV). After about 40 s, the first phase was followed by the almost complete disappearance of spontaneous action potentials (Fig. 5a(3),b(3)). Steady-state voltage-clamp experiments performed at a holding potential of −50mV revealed that IR3535 (100 nM), in the presence of pirenzepine (100 nM), produced an inward current, corresponding to the current previously described (Fig. 3d), followed by the development of an outward current (Fig. 5c(3)). This outward current was strongly reduced in the presence of TEA-Cl (10 mM) or when the steady-state holding potential was hyperpolarized to −100mV, a value close to the calculated Nernstian equilibrium potential for potassium ions (−100.8 mV), in our experimental conditions (Fig. 5d(3)). In addition, bath application of IR3535 (100 nM) in the presence of pirenzepine (100 nM) was tested on the input membrane resistance under current-clamp in response to a hyperpolarizing current pulse (100 ms in duration; Fig. 5e,f). The input membrane resistance was increased by 32.0 + 9.8% (n = 7) after IR3535 treatment (Fig. 5e(2)) and then was decreased by 14.8 + 6.3% (n = 6) (Fig. 5f(3)). These results indicate that the development of both inward and outward currents, assumed to be carried by potassium ions through background channels, correlated well with i) the IR3535-induced increase and decrease in the input membrane resistance, respectively and ii) the action potential discharge frequency increased followed by the disappearance of spontaneous action potentials (Fig. 5a–f). Interestingly, bath application of IR3535 (100 nM), in the presence of higher concentration of pirenzepine (i.e., 500 nM), only produced a slight hyperpolarization (2–3 mV) associated, within the first 30 s, with a strong reduction of action potential discharge frequency (Fig. 5g(3),h(3)). We never observed the first effect reported with lower concentration of pirenzepine (i.e., 100 nM). These results seem to indicate that high concentration of IR3535 (100 nM) was capable of displacing 100 nM pirenzepine, resulting in the dual effect as it is proposed in Fig. 5a,j. By contrast, increased concentration of pirenzepine (i.e., 500 nM) allowed to only reveal the second action of IR3535 (Fig. 5i). The experiments performed in the presence of pirenzepine (100 nM) unmasked an additional effect of IR3535 on M1-mAChR. One of the reason explaining why IR3535 (100 nM) elicited inward and outward potassium currents is that pirenzepine used at 100 nM could be displaced from the M1-mAChR orthosteric site by higher concentration of IR3535 (100 nM), depending on the time of application. This results in the generation of the inward potassium currents followed by the development of the outward current, which is consistent with a possible interaction of IR3535 with another M1-mAChR site, such as for instance an allosteric site. Pirenzepine used at higher concentration allows to only reveal the effect of IR3535 on a putative different site (Fig. 5i).

**Figure 6:**
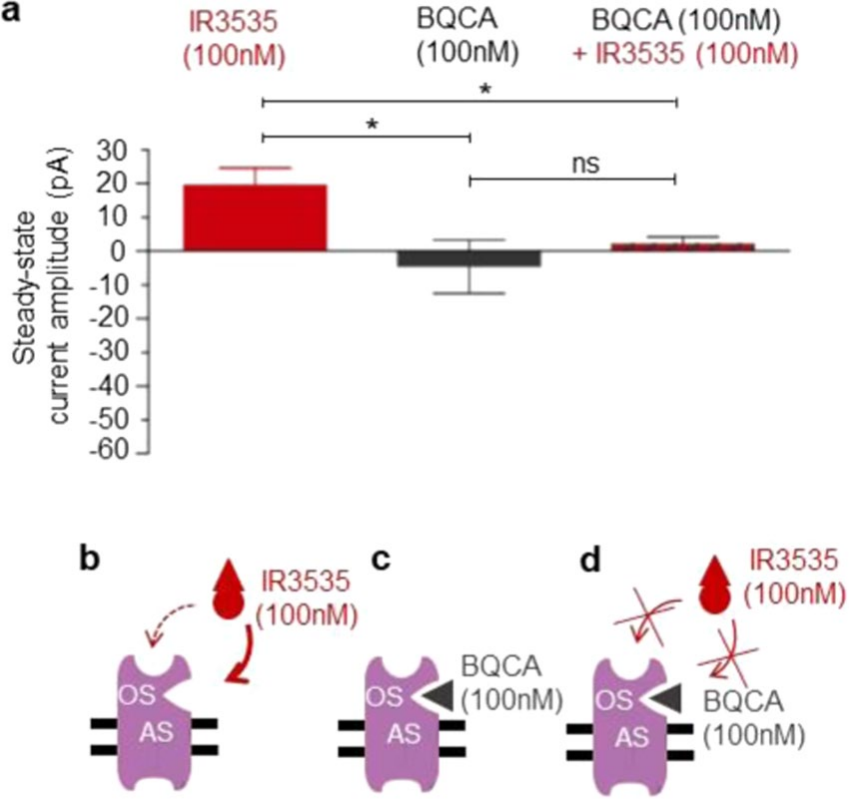
High concentration of IR3535 interacts with the allosteric site on M1-mAChR. (**a**) Comparative histogram showing the inhibition of the outward potassium current, recorded under voltage-clamp at a holding potential of −50mV, induced by IR3535 (100 nM) by pretreatment with the well known M1-mAChR allosteric modulator BQCA (100 nM). In the absence of orthosteric site agonist, BQCA (100 nM) applied alone has no significant effect. It is interesting to note that in the presence of BQCA, IR3535 (100 nM) never generates inward current, reflecting the activation of orthosteric site. Average data is shown as mean ± S.E.M. (n = 5–7), statistical test is Student unpaired t-test *p < 0.05; ns, non significant. (**b–d**) Hypothetic schemes illustrating that i) the high selective allosteric modulator of M1-mAChR, BQCA completely inhibits the outward current induced by IR3535 (100 nM) and ii) high concentration of IR3535 (100 nM) could also interacts with a different site than the M1-mAChR orthosteric site on M1-mAChR, reinforcing the involvement of allosteric binding site in the complex effect of IR3535.

**Figure 7:**
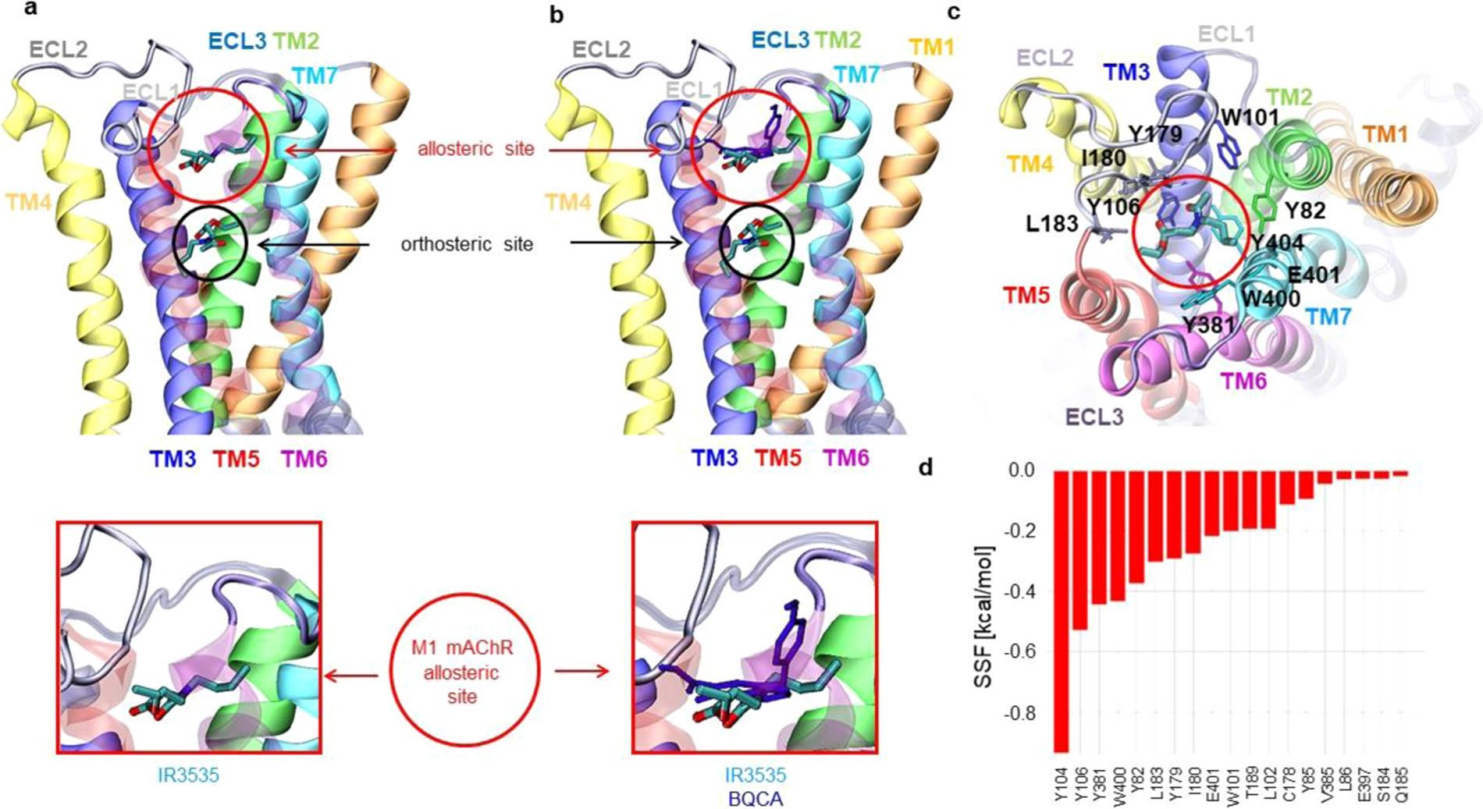
Computational docking results of ligands interactions at the allosteric site of M1 mACh recepto**r**. The highest affinity poses of: IR3535 (red circle) (**a**) and BQCA (blue sticks in red circle) (**b**) located at the allosteric site of M1-mAChR. In panel (a,b) the pose of IR3535 at the orthosteric site is also displayed for clarity (below red circle). The mean value and standard deviation of binding energy has been calculated for each ligand based on the five runs. The energy of binding is: (−5.1 ± 0.1) kcal/mol for IR3535 and (−8.5 ± 0.1) kcal/mol for BQCA, respectively. (**c**) Close-up view of the highest affinity pose of IR3535 from the top view of M1-mAChR. Residues observed in the close vicinity of IR3535 are displayed in stick representation and coded by the color of transmembrane helices: TM1 - orange, TM2 - green, TM3 - dark blue, TM4 - yellow, TM5 - red, TM6 - magenta, TM7 - light blue. (**d**) Residues with the highest contribution to the energy of binding of IR3535 at the allosteric site of M1-mAChR.

To check whether high concentration of IR3535 interacted with a completely different site than M1-AChR orthosteric site, experiments were performed in the presence of BQCA (100 nM), a highly selective allosteric modulator of the M1-mAChR^33,34^ (Fig. 6a). If the allosteric modulator applied alone did not produce any agonist activity (Fig. 6a,c), pretreatment with BQCA prevented the action of 100 nM IR3535 (Fig. 6a,d). These last results strongly suggest that at high concentration IR3535 molecules could also bind to a different site than the M1- mAChR orthosteric site (Fig. 6b). To further support this idea, computational docking of IR3535 and BQCA interactions at the allosteric site of M1-mACh receptor was performed realized. In Fig. 7a–c, the M1-mAChR allosteric site for IR3535 was displayed. The IR3535 ligand in the allosteric site was less buried than in the orthosteric one (Fig. 4a *versus* 7a), therefore the total contribution to the energy of binding coming from the allosteric site residues located within 6 Å distance to the ligand was smaller than before. A strong interaction involved a slightly smaller number of residues (Y104, Y106, Y381, W400) contributing substantially to the binding (SSF > −0.4 kcal/mol) (Fig. 7d). The conformations of IR3535 docked to the allosteric site were compared to the highly selective allosteric modulator of the M1-mAChR, BQCA (Fig. 7b, blue sticks). The allosteric site best poses were similar for IR3535 and BQCA but the binding energy was significantly higher for BQCA. All together, these results indicate that IR3535, is able to mediate M1-mAChR activation in its own right by interacting to a recognition region in the M1-mAChR that is distinct from the primary, orthosteric site.

### Allosteric site stimulation modifies ligand interaction with orthosteric site of M1-mAChR in DUM neurons

**Figure 8:**
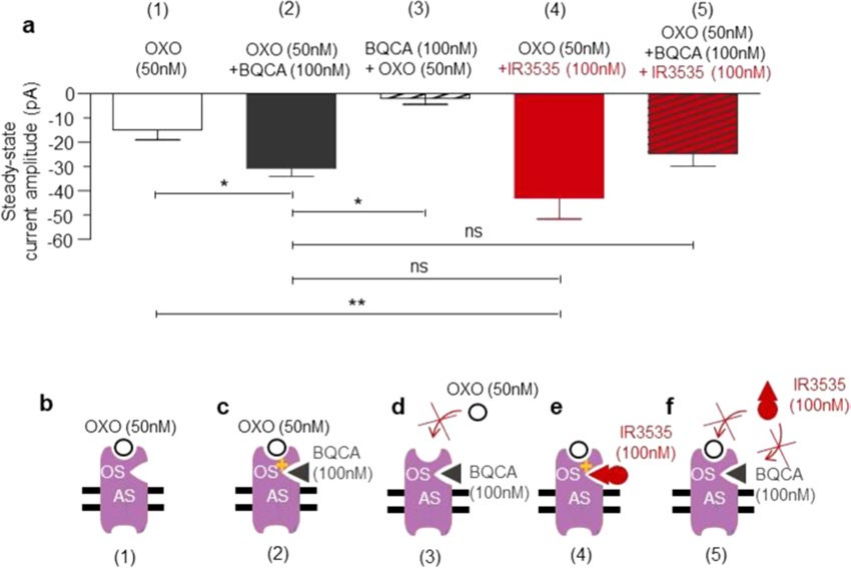
Allosteric site activation limits ligand effect with orthosteric site of M1-mAChR in DUM neurons. (**a**) Comparative histogram illustrating the inward current amplitude generated by the well known M1-mAChR agonist oxotremorine-M (OXO) and IR3535 recorded under voltage-clamp at a holding potential of −50mV. Number in brackets indicated above each bar represent the different experimental conditions. (**b–f**) Hypothetic models based on the results obtained in (**a**). Number in brackets mentioned below each schemes correlates well with those indicated in (**a**). Average data is shown as mean ± S.E.M. (n = 3–11); statistical test is Student unpaired t-test, **p < 0.01; *p < 0.05; ns, non significant.).

From the data presented just above, an important question still has to be answered regarding the development of the outward current only observed with high concentration of IR3535 (i. e.,100 nM), which follows the early inward potassium current (Figs. 5c(3), 6a). Based on results illustrated in Figs. 6a,b,d and 7, interaction with M1-mAChR allosteric site may induce a conformational change of orthosteric site, limiting the effects of IR3535 (Fig. 6d). In other words, IR3535 used at high concentration could be also considered as an allosteric modulator of the function of the orthosteric agonist, revealing this unexpected outward current. Consequently, additional set of experiments were designed to understand better this non-classical allosteric mechanism (Fig. 8).

Under voltage-clamp at a holding potential of −50mV, application of oxotremorine-M (50 nM), the M1-mAChR agonist, induced, as expected, an inward current reflecting the agonist effect on the orthosteric site, previously described (Figs. 3d,e, 8a,b). Then, experiments were performed with BQCA, the highly selective positive allosteric modulator for M1-mAChR (see also Fig. 7b). BQCA (100 nM) potentiated the oxotremorine-M-induced inward current to nearly 200% of control (Fig. 8a,c). By contrast, when DUM neuron cell body was pretreated with BQCA (100 nM), oxotremorine-M (50 nM) was not able to produce any inward current (Fig. 8a,d). Similar experiments were performed with IR3535 (100 nM). As shown in Fig. 8a,e, IR3535 also enhanced the inward current amplitude generated by oxotremorine-M (50 nM) to nearly 290%. We next tested IR3535 (100 nM) while maintaining M1-mAChR allosteric site activated by BQCA (100 nM). In this condition, the oxotremorine-M-induced current amplitude was very similar to that of recorded with BQCA (Fig. 8a,c,f). We never observed any additional effect produced by IR3535 (100 nM). These results confirm that IR3535 used at relatively high concentration might also be considered as a M1-mAChR allosteric modulator. The potentiating effect is only observed when the agonist is present. By contrast, the lack of effect of IR3535 (100 nM) on orthosteric site after pretreatment with BQCA, suggests that interaction with M1-mAChR allosteric site may modify conformation of the orthosteric site limiting the effect of agonist (Fig. 6d).

### Synergistic interactions between IR3535 and the neonicotinoid insecticide, thiacloprid

**Figure 9:**
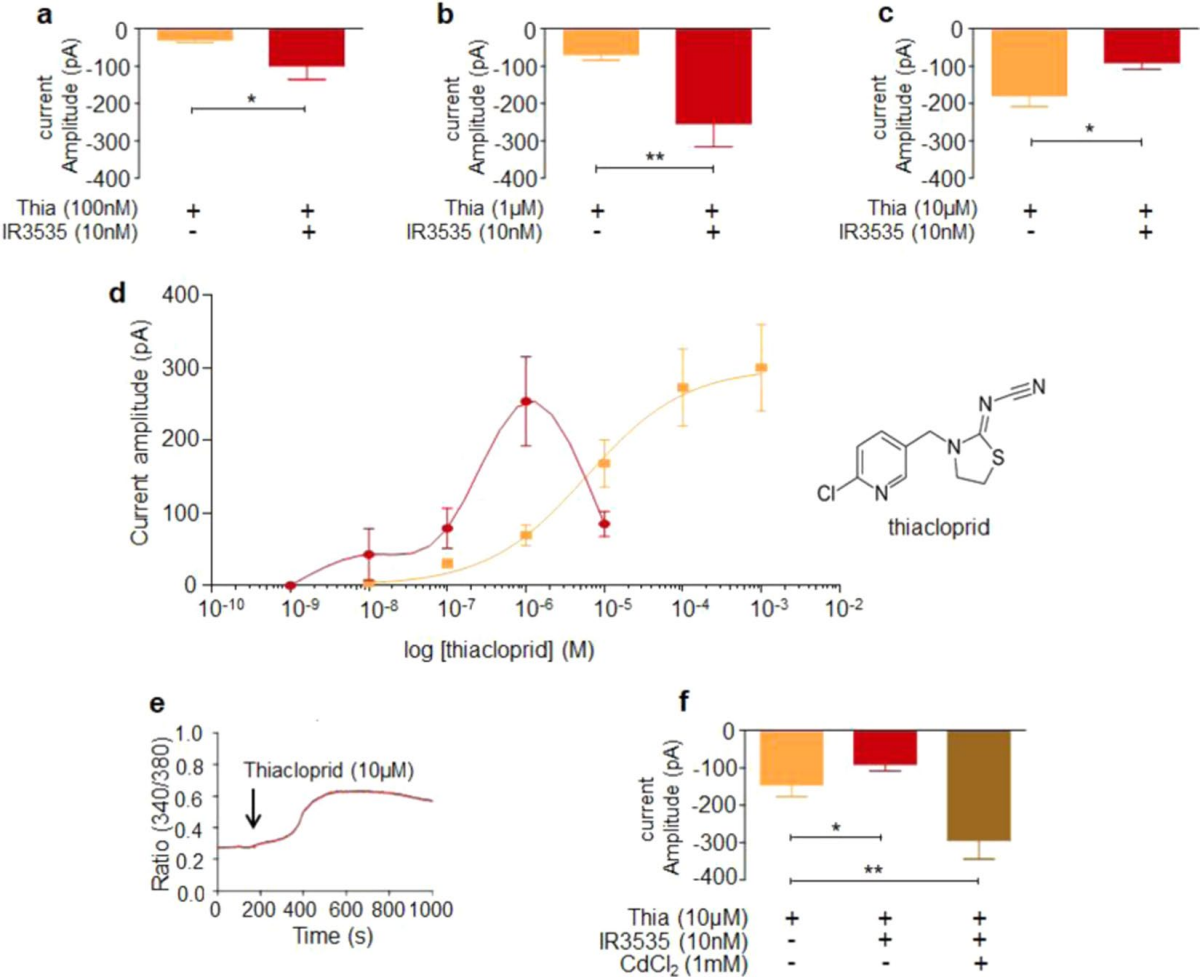
Low concentration oncentration of IR3535, used as synergistic agent, increases the effect of thiacloprid, a neonicotinoid insecticide. (**a,b,c**) Histograms illustrating the concentration-dependent amplitudes of the thiacloprid-induced inward currents recorded under voltage-clamp conditions at a steady-state holding potential of −50 mV, applied alone at 100 nM (**a**), 1 µM (**b**) and 10 µM (**c**) and after pretreatment with 10 nM IR3535. Note that IR3535 significantly potentiates the amplitude of the current produced by thiacloprid (**a,b**) whereas it is strongly decreased for high concentration of thiacloprid (**c**). Data are means ± S.E.M. (n = 4–7), statistical test is Student unpaired t-test *p < 0.05; **p < 0.01. (**d**) Superimposed semi-logarithmic concentration-response curves for the inward current induced by thiacloprid applied alone and in the presence of 10 nM IR3535. Inset represents the chemical structure of the neonicotinoid insecticide, thiacloprid. (**e**) Bath application of 10 µM thiacloprid increases intracellular calcium concentration in Fura-2 loaded DUM neuron cell body. (**f**) Histogram illustrating that CdCl_2_ (1 mM), an inorganic calcium channel blocker counteracts the effect of IR3535 (10 nM) applied in combination with high concentration of thiacloprid (10 µM). Data are means ± S.E.M. (n = 3–9), statistical test is Student unpaired t-test, *p < 0.05; **p < 0.01.

We then focused our study on the potential use of IR3535 as synergistic agent^24^ to potentiate insecticide effect while reducing the concentration. It is known that DUM neuron display membrane potential properties regulated by the activation of nicotinic acetylcholine receptors (nAChRs). Among insecticides acting on nAChRs, neonicotinoids are known to mimic the action of acetylcholine by targeting insect nAChRs^35–37^. We tested, under voltage-clamp, the effect of thiacloprid, a cyanoimine of the neonicotinoid class (Fig. 9d; inset), at a steady-state holding potential of −50 mV. Isolated DUM neurons were exposed to various concentrations of thiacloprid (Fig. 9a,b,c,d). Mean values of the thiacloprid-induced inward current amplitude were plotted against the logarithm of the non-cumulative concentration of thiacloprid (Fig. 9d). The sigmoid curve corresponded to the best fit through the mean data points (correlation coefficient r = 0.994) according to the Hill Eq. (1). The EC_50_ value estimated for thiacloprid (the concentration of thiacloprid that produces 50% of the inward current amplitude) was 5.8.10^–6^M (Fig. 9d). In the presence of IR3535 (10 nM), we observed a non-monotonic concentration-effect curve, shifted to the left (Fig. 9d) with the maximum steady-state inward current amplitude significantly increased from 10^–8^M to 10^–6^M compared to the current amplitude induced by thiacloprid alone (Fig. 9a,b,d). Interestingly, the inward current amplitude reached a maximum at 10^–6^M before decreasing for higher concentrations of IR3535 than 10^–6^M (Fig. 9c,d). The potentiation of the inward current amplitude induced by thiacloprid (100 nM) in the presence of IR3535 (10 nM) was abolished after pretreatment with pirenzepine (100 nM, not shown). We previously reported that elevation of the [Ca^2+^]_i_ is now known to be the first step of molecular events involved for increasing the sensitivity to insecticides^23,24,38^. In this study, we revealed that IR3535 (10 nM), elevating the [Ca^2+^]_i_ *via* M1-mAChR orthosteric site activation (Figs. 1 and 2), potentiated the effect of thiacloprid but only at concentrations ranging from 10^–8^M to 10^–6^M (Fig. 9d). Because calcium influx through calcium channels regulates nAChR functions^39,40^ and modulates the sensitivity of nAChRs to neonicotinoid insecticides^23,35^, the biphasic aspect of the concentration-response curve observed for high concentrations of thiacloprid suggests that an important increase in [Ca^2+^]_i_ could reduce the sensitivity of nAChRs to thiacloprid. We therefore examined the effect of thiacloprid (10 µM) on [Ca^2+^]_i_ (Fig. 9e). Bath application of 10 µM thiacloprid produced a marked elevation in [Ca^2+^]_i_ followed by a sustained elevated level. Parallel experiments performed with the calcium channel blocker CdCl_2_ (1 mM) revealed an important increase in the steady-state inward current amplitude produced by thiacloprid (10 µM) in the presence of IR3535 (10 nM; Fig. 9f).

## Discussion

Despite the numerous studies that have been carried out on the mode of action of insect repellents over the past decades^41^, much questions remain. Here we show that IR3535 displays complex effects on neuronal M1-mAChRs. Although M1-mAChR and more generally GPCRs are not considered as key targets used for insect control, some results begin to highlight the importance of these receptors as exciting elements in the development of novel strategies against insects^42,43^. In this context, insects M1/M3-mAChRs have already been reported to play fundamental role in the synergy between the repellent DEET and the carbamate insecticide, propoxur *via* calcium-dependent intracellular signalling pathways, extending efficacy of the treatment^18^. These results opened new fields of investigation regarding the importance of both mAChRs and the insect repellents, through an unexpected mode of action to control insects. They also emphase that insect repellents such as IR3535 (this study) could not be used only as classical repellent but as a synergistic agent, which is an emerging concept to optimise insecticide treatment against insects^24^.

**Figure 10:**
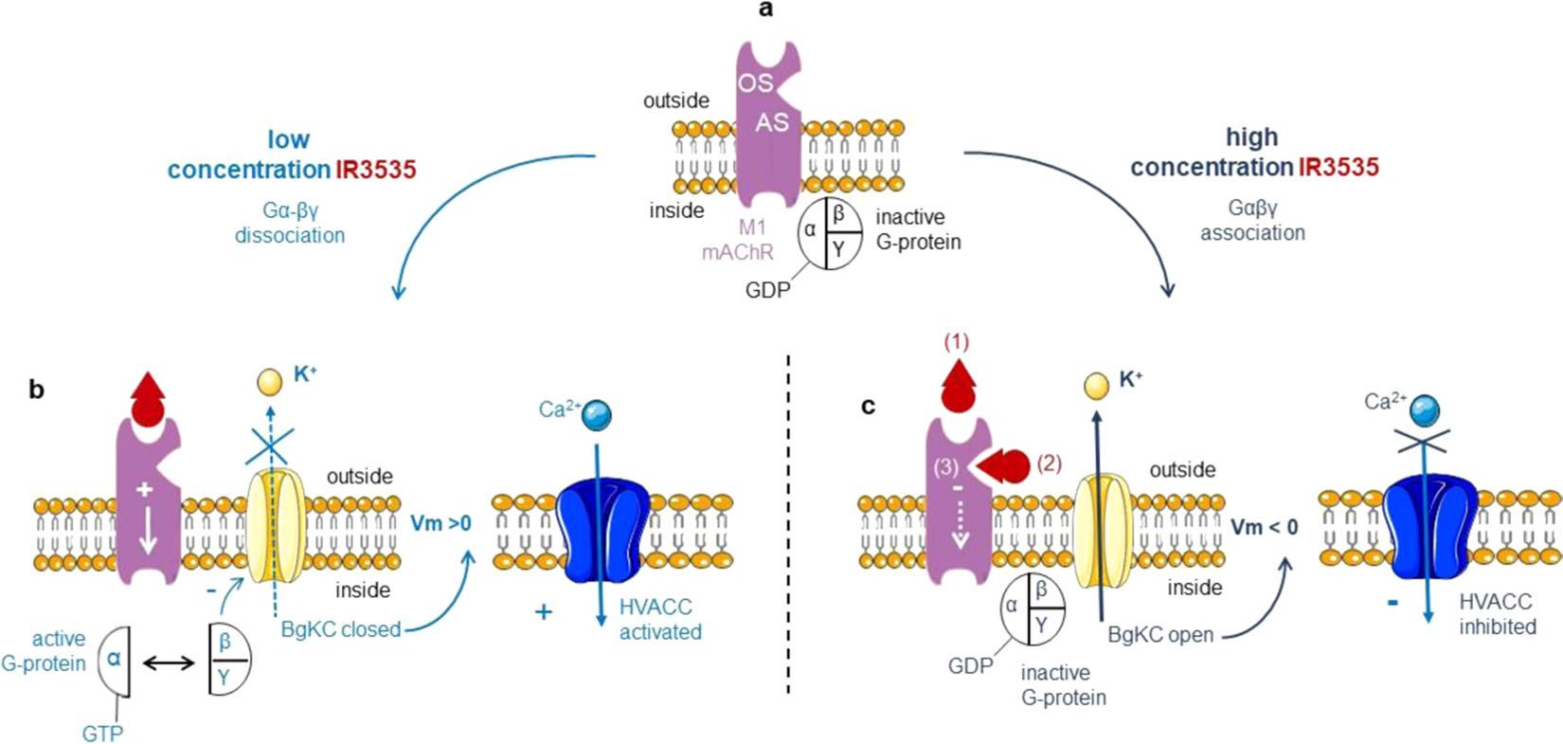
Diagram representing the molecular events involved in the activation of M1 muscarinic acetylcholine receptor by IR3535. (**a**) IR3535, depending on the concentration tested, binds to distinct sites (M1-mAChR orthosteric and allosteric sites) that couple to the heterotrimeric G protein underlying the signal transduction that leads to IR3535-induced modulation of BgKC. (**b**) At low concentration, IR3535 interacts with M1-mAChR orthosteric site, which stimulation leads to trigger Gαβγ dissociation. The release of Gβγ subunits inhibits background potassium current, resulting in membrane depolarization, which thereby activates high-voltage-activated calcium channels governing calcium influx. (**c**) At higher concentration, IR3535 induces dual effect on both M1-mAChR orthosteric and allosteric sites, depending on the time of exposure. Within the first seconds, IR3535 stimulates the orthosteric site (1) before interacting with the allosteric site (2). This last effect renders the G protein inactivated (3), which maintains the BgKC functional resulting in the hyperpolarized potential where high-voltage-activated calcium channels remain closed. Vm > 0 and Vm < 0 mean depolarization and hyperpolarization, respectively. M1-mAChR, M1 muscarinic acetylcholine receptor subtype; HVACC, high-voltage-activated calcium channels; BgKC, background potassium channels; OS and AS orthosteric and allosteric sites, respectively.

Except previous findings reporting that IR3535 affects the function of odorant receptors and gustatory sensitivity of insects^20,44–46^, the neurophysiological mechanism of IR3535 still remain to be investigated. Using a multidisciplinary approach, we have established that multiple mechanisms of action explain complex IR3535 activity in insect neurosecretory cells, *via* the interaction with the M1-mAChR (Fig. 10a). The IR3535-induced intracellular calcium rise is strongly correlated with interaction on M1-mAChR orthosteric site, an effect depending on the concentration of IR3535 tested. As summarized in Fig. 10b, low concentration of IR3535, (i.e 10 nM) activates the orthosteric site resulting in the dissociation of heterotrimeric G protein into their component Gα and Gβγ subunits. We have established an independent role in signaling for Gβγ subunits distinct from Gα in the direct inhibition of the BgKC involved in the maintenance of the membrane potential of DUM neurons^25,27^. In this case, Gβγ are considered as direct negative regulators of the potassium channels. The inhibiting effect of Gβγ on the BgKC generates membrane depolarization, which activates HVA calcium channels and calcium influx. This atypical mechanism expands the physiological role of Gβγ subunits already reported in cells^28,47^. One of the most interesting feature is the unexpected bell-shaped concentration-effect relationships of IR3535 observed for the very low concentration range (i.e., between 1 nM and 100 nM). Unlike standard sigmoidal curves, bell-shaped concentration–response curve suggests more complex biological effects such as, for instance, multiple-binding sites. IR3535 increases intracellular calcium level at low concentration (10 nM) but inhibits calcium rise at higher concentrations (100 nM). In this last case, the inhibition of the elevation of [Ca^2+^]_i_ may be explained by the hypothetical model illustrated in Fig. 10c. During the period of time of the treatment by 100 nM IR3535, the dual effect observed involves two different sites. At the beginning of the treatment, the effect of IR3535 on the M1-mAChR orthosteric site generates the early inward potassium current (Fig. 10c(1)). Then after, IR3535 interacts with an allosteric site, distinct from the orthosteric binding site, that leads to BgKC activation and membrane hyperpolarization (Fig. 10c(2)). Interestingly, in the presence of oxotremorine-M, IR3535 (100 nM) increases the activity of the agonist, like the positive allosteric modulator BQCA. However, this effect disappears as exposure time increases, revealing the second action described just above. This last pharmacological action contrasts with the classical allosteric mechanisms^48,49^. It seems that this interaction allosterically influences the ligand binding on the orthosteric site, which in turn decreases the effect of IR3535. In this case the partial agonist effect of IR3535 stabilizes the receptor in its active conformation less effectively, which do not engage conformational changes leading to the dissociation between Gα and Gβγ subunits. The G protein heterotrimers remain intact, keeping the BgKC open governing membrane hyperpolarization and inhibition of calcium influx (Fig. 10c(3)). This is confirmed by the experiments illustrated in Supplementary Fig 2 indicating that IR3535 (100 nM) never produces significant outward potassium current when the experiments were performed with GTP-γ-S, a non-hydrolyzable G-protein-activating analog of GTP maintaining G protein activation. Based on these results, IR3535 is believed to produce either inhibition or activation of BgKC through modulation of M1-mAChRs. Depending on the concentration tested, IR3535 produces distinct effect manifested as a switch from inhibition to activation of potassium channels. This mechanism appears to involve a cross-talk between allosteric and orthosteric sites of the active receptor conformation explaining both the increase and the decrease in the action potential discharge frequency observed during time of IR3535 exposure (see Fig. 5a,b). Although M1-mAChRs are targeted by IR3535, the effects reported in this study vary somewhat from those characterized with another repellent, DEET^18^. The maximum intracellular calcium rise observed is reached at a concentration 10-fold lower than with DEET and the intracellular signalling pathways following the interaction of IR3535 with M1-mAChRs differ from those previously described with DEET in the same neuronal preparation. This reinforced the importance of obtaining deeper knowledges on the multifaceted effects of repellent in insects.

As previously stated, the aim of this study is also to envisage the use of IR3535 at low concentration as synergistic agent to optimize insecticide efficacy. It is now accepted that calcium-dependent intracellular regulation (i.e. phosphorylation/dephosphorylation process) increases plasma membrane receptors and/or ion channels sensitivity to insecticides^18,23,50^. In this context, the use of repellent and insecticide co-formulated mixtures is a topic of great interest currently, particularly in the integrated vector management (IVM)^18,24,51–53^. Here, we report the development of an innovative vector control strategy using IR3535. However, the level of the intracellular calcium rise must be kept within a strictly controlled range to avoid opposite effects. Because of their crucial role in triggering rapid neural transmission, nAChRs are the primary target site of a broad range of insecticides including neonicotinoids^36,37,54,55^. We have demonstrated in this study that only low concentration of IR3535 (10 nM) increases nAChR sensitivity to the neonicotinoid insecticide thiacloprid *via* the internal calcium rise. The significant shift of the sigmoid curve towards lower insecticide concentrations associated with an increase of the thiacloprid effect confirm the important role of the synergistic agent (IR3535) in the optimization of the insecticide action, obtained at lower concentration, when compared to the effect of the insecticide applied alone. However, as indicated above, the synergism observed is very dependent on the [Ca^2+^]_i_ elevation. We have shown that relatively high concentration of thiacloprid, applied alone, also increases [Ca^2+^]_i_. IR3535 combined with high concentration of thiacloprid results in a strong decrease of nAChR sensitivity to this insecticide. This opposite effect reflects the important role of calcium allosteric modulation involved in the regulation of insect pharmacological nAChR activity. Furthermore, we also reveal that relatively high concentration of IR3535 (i.e., 100 nM) through its interaction with the M1-mAChR allosteric site, is not able to produce any intracellular calcium rise, a key element in the synergism between IR3535 and thiacloprid. This confirms that it was essential to characterize precisely the mode of action of IR3535 before its use as a synergistic agent. The synergism observed is only possible in the narrow low concentration range. IR3535 is nevertheless able to trigger intracellular calcium rise, thereby activating a calcium-dependent signalling involved in the optimization of the nAChR sensitivity to thiacloprid.

In conclusion, the synergistic interaction between IR3535 and thiacloprid contributes to significantly increase the efficacy of the treatment while reducing concentrations. This is particularly in line with the recommended safe use of phytosanitary compounds in IVM.

## Methods

All experiments were performed on adult male cockroaches *Periplaneta americana* taken from our laboratory stock colony, maintained under standard conditions (29 °C, photo-cycle 12 h light/12 h dark).

### Cell preparation

Experiments were carried out on DUM neuron somata isolated from the midline of the terminal abdominal ganglion (TAG) of the nerve chord of adult male cockroaches (*Periplaneta americana*) as already described^56^. Animals were immobilized ventral side up on a dissection dish. The ventral cuticle and the accessory gland were removed to allow access to the TAG, which was carefully dissected under a binocular microscope and placed in normal cockroach saline containing (in mM): 200 NaCl, 3.1 KCl, 5 CaCl_2_, 4 MgCl_2_, 10 HEPES, and 50 sucrose, pH was adjusted to 7.4 with NaOH. Isolation of adult DUM neuron somata was performed under sterile conditions by using enzymatic digestion by collagenase (type IA, 280 IU/ml; Worthington Biochemicals, Freehold, NJ) at 29 °C for 35 min. Then, a mechanical dissociation through fire-polished Pasteur pipettes was used to isolate DUM neurons from the TAG^55^. DUM neuron somata were maintained at 29 °C for 24 h before electrophysiological experiments were carried out.

### Calcium imaging

DUM neuron cell bodies were isolated from the Terminal Abdominal Ganglion (TAG), as already mentioned above. The cells were washed two times in saline and incubated in the dark with 5 mM Fura-2 pentakis (acetoxy-methyl) ester (Fura-2 AM) (Sigma-Aldrich, Saint Quentin Fallavier, France) in the presence of 0.1% pluronic acid F68 (Sigma- Aldrich, Saint Quentin Fallavier, France) for 1 h at 37 °C. Pluronic acid is a nonionic surfactant used as a stabilizer of cell membrane protecting from membrane shearing to facilitate uptake of Fura-2 AM. After loading, cells were washed two times in saline. The glass coverslips were then mounted in a recording chamber (Warner Instruments, Hamden, CT, USA) connected to a gravity perfusion system allowing drug application. Imaging experiments were performed with an inverted Nikon Eclipse Ti microscope (Nikon, Tokyo, Japan) equipped with epifluorescence. Excitation light was provided by a 75-W integral xenon lamp. Excitation wavelengths (340 nm and 380 nm) were applied using a Lamdba DG4 wavelength switcher (Sutter instrument, Novato, CA, USA). Images were collected with an Orca-R2 CCD camera (Hamamatsu photonics, Shizuoka, Japan) and recorded on the computer with Imaging Workbench software (version 6, Indec BioSystems, Santa Clara, CA, USA). Experiments were carried out at room temperature. Intracellular calcium level was expressed as the ratio of emitted fluorescence (340/380 nm), as previously reported^57^.

### Electrophysiology and data analysis

The patch-clamp technique in the whole-cell recording configuration was used to record spontaneous action potentials and input membrane resistances (current-clamp mode) and steady-state ionic currents (voltage-clamp mode)^35,56–58^. Signals were recorded with an Axopatch 200 A patch-clamp amplifier (Axon instruments), digitized and acquired using a MiniDigidata 1440 analog-digital converter (Axon Instruments). Currents were treated with axo- scope 10.2 software (Axon Instruments). Patch pipettes were pulled from borosilicate glass capillary tubes (GC 150T-10; Clark Electromedical Instruments, Harvard Appartus Edenbridge, UK) using a P-97 Flaming/Brown Micropipette Puller (Sutter Instrument Company, Novato, U.S.A). Pipettes had resistances ranging from 1 to 1.5 MΩ when filled with internal pipette solution (see composition below). The liquid junction potential between bath and internal solutions was always corrected before the formation of a gigaohm seal (>1 GΩ). Steady-state ionic currents induced by IR3535 and thiacloprid were recorded with software control pClamp (version 10.1; Axon instruments) and were low-pass filtered at 10 kHz with clampfit software (version 10.1; Axon instruments). Experiments were carried out at 20 °C.

We performed data analysis including fitting procedures, by using the software Prism 5.0 (GraphPad Software, Inc., La Jolla, CA, USA). To estimate the EC_50_ value of the curve fitting, data were analyzed with the GraphPad Prism version 5.0 (GraphPad Software, La Jolla, CA, USA). The Hill equation used to fit the sigmoid curve was:

1 $${\rm{Y}}={\rm{B}}{\rm{o}}{\rm{t}}{\rm{t}}{\rm{o}}{\rm{m}}+({\rm{T}}{\rm{o}}{\rm{p}}-{\rm{B}}{\rm{o}}{\rm{t}}{\rm{t}}{\rm{o}}{\rm{m}})/(1+{10}^{\wedge }(({\rm{L}}{\rm{o}}{\rm{g}}{\rm{E}}{{\rm{C}}}_{50}-{\rm{X}})\ast {\rm{H}}{\rm{i}}{\rm{l}}{\rm{l}}{\rm{S}}{\rm{l}}{\rm{o}}{\rm{p}}{\rm{e}}))$$

where Top and Bottom are plateaux in the units of the Y axis, EC_50_ is the concentration that gives halfway between Bottom and Top. HillSlope describes the steepness of the curve.

For statistical analysis, data are presented as the mean ± S.E.M. Information regarding which pairs of means were significantly different and which were not was determined by Student’s *t* test for multiple comparisons. In this case, statistical analysis was expressed as nonsignificant for *p* > 0.05 and significant for *p < 0.05, **p < 0.01, and ***p < 0.001.

### Solution and drug applications

Bath solution superfusing the cells contained (in mM): 200 NaCl, 3.1 KCl, 5 CaCl_2_, 4 MgCl_2_, and 10 HEPES; pH was adjusted to 7.4 with NaOH^35^. Patch pipettes were filled with solution containing (in mM): 160 K/Dgluconate, 10 KF, 10 NaCl, 1 MgCl_2_, 0.5 CaCl_2_, 1 ATP; 0.1 cAMP, 10 EGTA, and 10 HEPES; pH was adjusted to 7.4 with KOH. Thiacloprid stock solutions (1 M) and BQCA, IR3535 and gallein stock solutions (10^−2^M) were prepared in DMSO and then diluted in the bath solution to obtain the different concentrations tested. The highest concentration used in the electrophysiological recordings of DMSO was 0.1%. This concentration of solvent was not found to have any effect on the electrophysiological properties of DUM neuron cell body. Pharmacological agents were applied by a gravity perfusion valve controller system (VC–6 M, Harvard apparatus, 1 s in duration) controlled by pClamp software (flow rate of perfusion: 0.5 ml/min). The perfusion tube was placed within 100 µm from the isolated neuron cell body. IR3535, BQCA, cadmium chloride, ω-conotoxin GVIA from *Conus geographus*, caffeine, TBM-8, pirenzepine, U73122 and TEA-Cl were added to the external solution. GDP-β-S was added in the internal pipette solution immediately before use. All compounds were purchased from Sigma Chemicals (L’isle d’Abeau Chesnes, France). Except gallein from R&D Systems-Bio-techne, (Lille, France), [D-Trp7,9,10]-substance P from Tocris Bioscience, (Bristol, UK) and IR3535 from Merck KGaA (Darmstadt, Germany).

### Molecular in silico docking

The molecular docking of ligands: IR3535, pirenzepine, oxotremorine-M and BQCA into a crystal structure (PDB code: 5cxv) of the excitatory ganglionic muscarinic acetylcholine receptor (M1-mAChR) was performed using SMINA^59^ package. The human M1 receptor structure was used for docking without modifications. The default SMINA scoring function (SSF) is derived from AutoDock Vina^60^ and includes three steric terms, a hydrogen bond term, a hydrophobic term, and a torsion count factor. Rigid docking was carried out with SMINA default settings; a grid of 38×32×44 Å^3^ size centered at 10 Å distance from the channel surface was used. The following protocol, called a “multiple molecular docking” (MMD), has been developed previously^61^ and used here to identify the preferred binding sites of a ligand in the M1 receptor: (i) For each ligand 5 independent SMINA docking runs were performed and the pose with the lowest SSF, i.e. having the highest affinity to M1-mAChR, was selected in each run. (ii) In the next step, the SMINA docking was performed again, but for the M1 receptor having this best pose occupied by the ligand. (iii) Again, the second best pose was determined using the procedure no. (iv) This cycle has been repeated until 10 best affinity binding poses were determined. (v) The MMD procedure was applied for all four ligands which resulted in 200 independent SMINA docking runs. The analysis and visualizations were made using the VMD code^62^ and home-made scripts in the ProDy^63^ package. The OS cavity volume was calculated using POCASA 1.1 server^32^.

## Supplementary information

Supplementary Information.
