# Supplementary figures and images for: Orthosteric muscarinic receptor activation by the insect repellent IR3535 opens new prospects in insecticide-based vector control
Source: Sci Rep. 2020 Apr 22;10:6842. doi: 10.1038/s41598-020-63957-x (PMC7176678; doi:10.1038/s41598-020-63957-x)

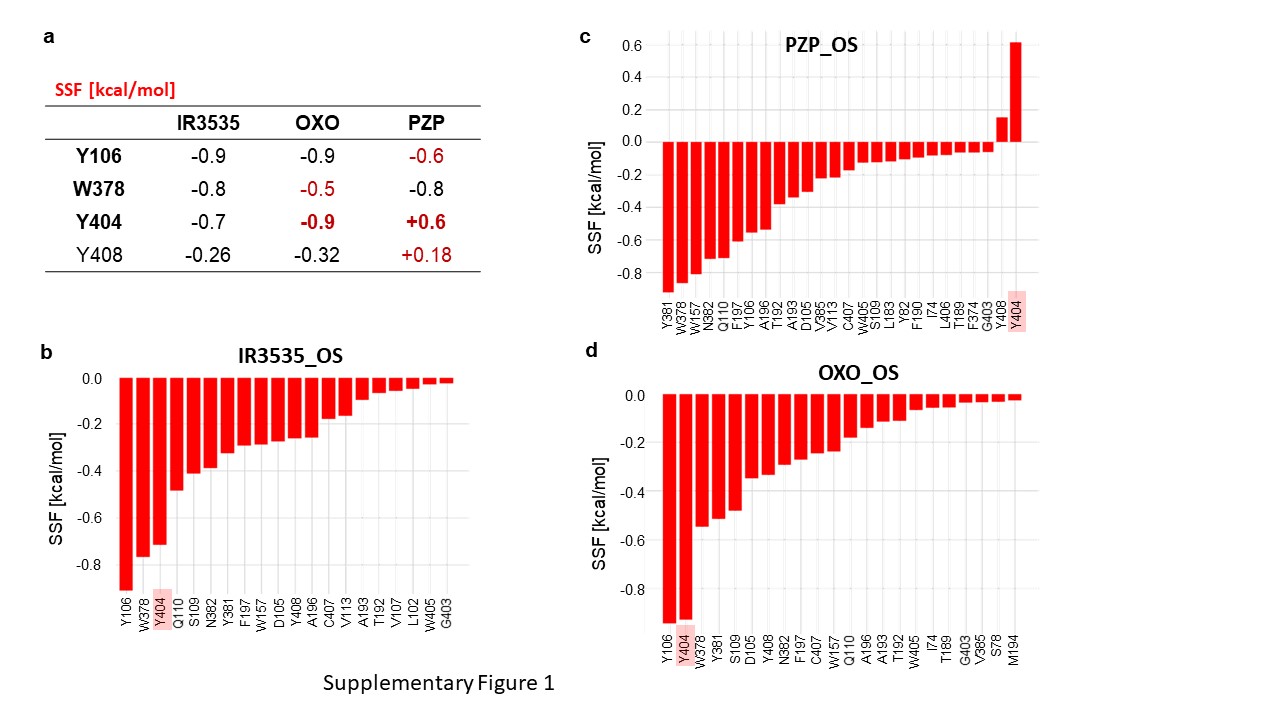


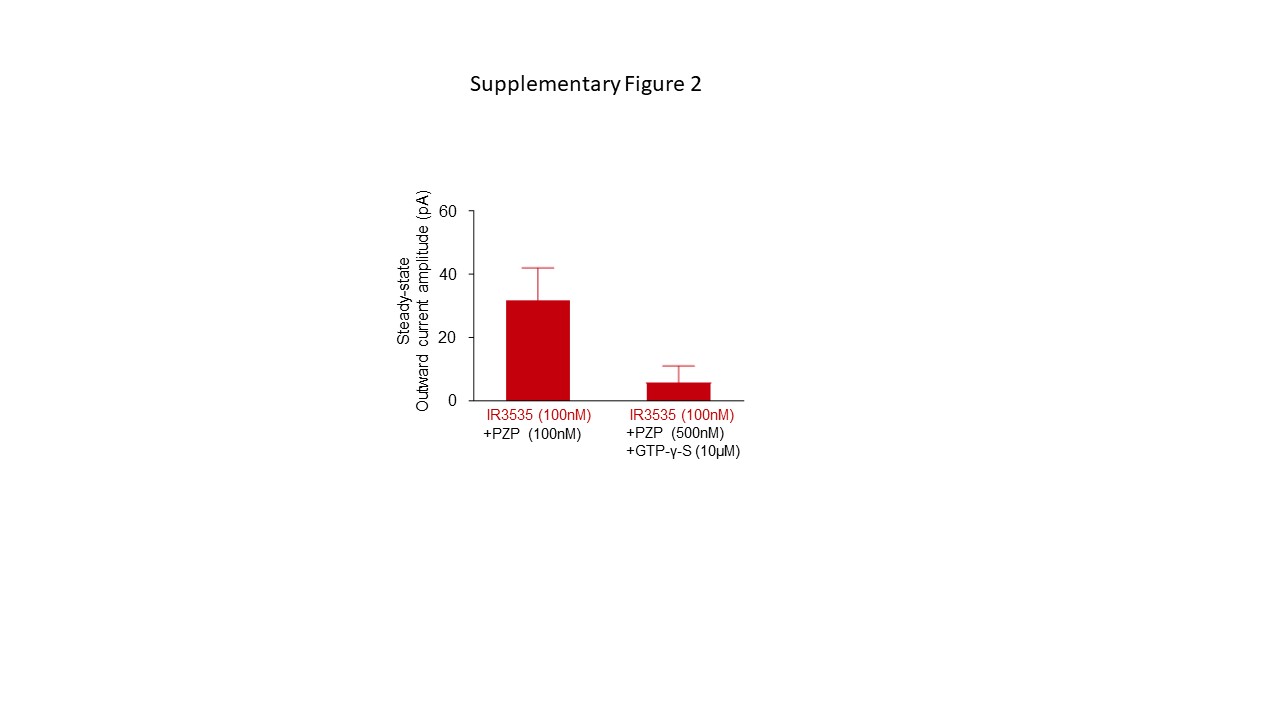

Supplement: Supplementary file 1 — Supplementary Information. [file 41598_2020_63957_MOESM1_ESM.docx]
